# Supplementary material for: Assessment of the prognostic value of CA125 for miscarriage risk in patients with threatened abortion: A systematic review and meta-analysis
Source: PLoS One. 2025 Jun 24;20(6):e0326384. doi: 10.1371/journal.pone.0326384 (PMC12186903; doi:10.1371/journal.pone.0326384)
Supplement: S1 File — (DOCX) [file pone.0326384.s002.docx]

**Original data**

| **No** | **Author** | **Year** | **TP** | **FP** | **FN** | **TN** |
| --- | --- | --- | --- | --- | --- | --- |
| 1 | Fahri | 1992 | 5 | 1 | 0 | 19 |
| 2 | Scarpellini | 1995 | 7 | 10 | 2 | 29 |
| 3 | Leylek | 1997 | 13 | 1 | 2 | 24 |
| 4 | Sherif | 2000 | 41 | 1 | 2 | 56 |
| 5 | Fiegler | 2003 | 66 | 18 | 6 | 110 |
| 6 | Maged | 2013 | 52 | 19 | 13 | 66 |
| 7 | Xie | 2014 | 72 | 9 | 7 | 47 |
| 8 | Sweed | 2016 | 23 | 3 | 7 | 87 |
| 9 | Maged | 2016 | 19 | 0 | 1 | 80 |
| 10 | Mansy | 2017 | 8 | 7 | 1 | 29 |
| 11 | Nesreen | 2018 | 14 | 16 | 2 | 68 |
| 12 | Mosunmola | 2019 | 10 | 8 | 5 | 40 |
| 13 | Mohamed | 2020 | 9 | 1 | 0 | 30 |

**Packages used in STATA program**

Packages：ssc install midas

ssc install mylabels

Sensitivity、Specificity：midas tp fp fn tn,res(all)

publication bias：midas tp fp fn tn,pubbias

Forest plot：midas tp fp fn tn, id(author year) ms(0.75) ford fors bfor(dss)

SROC curve：midas tp fp fn tn,plot sroc(both)

Meta-regression：midas tp fp fn tn,reg(1 2 3 4)

Likelihood ratio quadrant distribution plot：midas tp fp fn tn,lrmat
