# Supplementary material for: Assessment of the prognostic value of CA125 for miscarriage risk in patients with threatened abortion: A systematic review and meta-analysis
Source: PLoS One. 2025 Jun 24;20(6):e0326384. doi: 10.1371/journal.pone.0326384 (PMC12186903; doi:10.1371/journal.pone.0326384)
Supplement: Attachment — Submitted filename: Dear Editor.docx [file pone.0326384.s005.docx]

**Dear Editor,**

Thank you for the opportunity to review the manuscript. This is an updated systematic from “Role of serum biomarkers in the prediction of outcome in women with threatened miscarriage: a systematic review and diagnostic accuracy meta-analysis”. I have some comments:

**Title**

- The term “pregnancy outcomes” is broad and could mean different things, such as miscarriage, preterm labor, or live birth rates. A more specific definition of the outcomes being assessed would make the title clearer.

**Abstract**

- The phrase "clinical value of serum CA125 levels" is not very precise. It would be assisted by specifying whether the focus is on the prediction of miscarriage, pregnancy outcomes in general, or a generalized diagnostic strategy.
- There is no discussion of why CA125 is being evaluated or its significance as a biomarker in threatened abortion. Briefly describing CA125's role in this context would improve accessibility for readers unfamiliar with the topic.
- The conclusion claims “high diagnostic value” for CA125 but does not link this to clinical decision-making or how it could change current practice. Adding a practical implication would strengthen the conclusion.
