# Supplementary material for: Assessment of the prognostic value of CA125 for miscarriage risk in patients with threatened abortion: A systematic review and meta-analysis
Source: PLoS One. 2025 Jun 24;20(6):e0326384. doi: 10.1371/journal.pone.0326384 (PMC12186903; doi:10.1371/journal.pone.0326384)
Supplement: Attachment — Submitted filename: Response to Reviewers.docx [file pone.0326384.s007.docx]

**Response to Reviewers' Comments**

We sincerely appreciate the reviewers' insightful comments and constructive suggestions, which have significantly improved the quality of our manuscript. Below, we provide a point-by-point response to each comment, detailing the revisions made to the manuscript.

Reviewer #1: I thank the respected editor and authors for the chance to review this interesting manuscript. The authors have aimed to investigate the value of CA125 in the prediction of the outcome of threatened abortion and have reported a possible prognostic value. There are a few major and minor points I would like to address:

**Major points:**

1.Considering that the authors have aimed at reviewing the predictive/prognostic value of CA125 for future events, if authors and editors see fit, the title of the manuscript could be changed to “Assessment of the prognostic value of CA125 for …”. A few sentencing changes should be made to the manuscript text to reflect this change. Keywords could also be adjusted.

**Response:：**We sincerely appreciate your valuable suggestion. As recommended, we have revised the title of the manuscript to:

**"Assessment of the prognostic value of CA125 for miscarriage risk in patients with threatened abortion—a systematic review and Meta-analysis"**

Additionally, we have carefully adjusted the relevant sentences throughout the manuscript to ensure consistency with the revised title. The keywords have also been updated.

We believe these modifications have improved the clarity and accuracy of our manuscript. Thank you for your constructive feedback.

2. Accordingly, using the QUAPAS (Quality Assessment of Prognostic Accuracy Studies) risk of bias assessment tool would be better suited in this manuscript, considering that this tool is designed explicitly for prognostic assessment studies while QUADAS tool was designed for diagnostic assessment studies.
QUAPAS Tool: Lee, Jenny, et al. "QUAPAS: an adaptation of the QUADAS-2 tool to assess prognostic accuracy studies." Annals of internal medicine 175.7 (2022): 1010-1018.

In addition, currently, authors have provided no explanations for the reasoning behind giving a judgment of high or unclear risk of bias. I suggest that after utilizing the QUAPAS tool, authors provide the reasonings behind their judgments of high or unclear risk of biases in any of the studies.

**Response:**
We sincerely appreciate this insightful suggestion. We fully agree that the QUAPAS tool is more appropriate for assessing prognostic accuracy studies in our systematic review. Following your recommendation, we have:

**1.Replaced the original QUADAS-2 assessment with the QUAPAS tool for all included studies.**

**2.Updated Figure 2 to present the new risk of bias assessment results using QUAPAS.**

**3.Provided detailed explanations for each judgment (high/unclear/low risk) in S3 File.**

The revised assessment has significantly strengthened the methodological rigor of our review. We are grateful for your expert guidance in improving this aspect of our work.

3. The authors have mentioned that “Diagnosis was assessed by combining SEN, SPE, PLR, NLR, DOR and AUC.”. Please further explain the analytic methodology, whether true positive, true negative, false positive, false negative values were extracted from articles or meta-analysis was somehow performed with SEN, SPE, PLR, NLR, DOR, and AUC values. Additionally, I suggest authors mention the packages used in STATA program, if any.

**Response:**

We sincerely appreciate the opportunity to clarify our methodology. As suggested, we have now provided more detailed explanations in the revised manuscript (Data extraction):

**In particular, when TP, FP, TN, FN, were not directly reported, we calculated them using the reported SEN and SPE values along with the sample size.**

The complete dataset including all extracted/calculated TP, FP, TN, FN values and packages used in STATA program have been provided in **S1 File.**

We believe these clarifications significantly improve the transparency and reproducibility of our analysis.

4. The utilized thresholds in studies are different. How was this issue addressed in the analysis, or were studies pooled together? If authors have pooled all analysis without considering the utilized cut-off, this could be more clearly mentioned in the methods or limitations section.

**Response:**

We sincerely appreciate this important methodological concern regarding varying CA125 thresholds across studies. As suggested, we have added the following to the results and Limitations section:

**1.results: studies were pooled despite varying CA125 cut-off values as threshold effect analysis demonstrated this was appropriate.**

**2.Limitations section:** We elucidate the differences in thresholds in depth in the discussion section of the revised manuscript (**Page 17, Lines300-304, Page 18, Lines308-315)**.

5. The authors could also add a summary of findings table and GRADE certainty of evidence assessment (Cochrane Systematic Review Handbook, Chapter 14: Completing ‘Summary of findings’ tables and grading the certainty of the evidence).

**Response:**

We sincerely appreciate this valuable suggestion to enhance the clinical utility of our systematic review. In response:
1. We have added a comprehensive **Summary of Findings (SoF) table** (now presented as Table 2 in the revised manuscript) that includes: **Outcome, Pooled Estimate (95% CI)	, GRADE Certainty, Factor of downgrade, Factor of escalation and Clinical Implications.**

2. The GRADE assessment was conducted following the Cochrane Handbook guidelines, considering: **Risk of bias, Inconsistency, Indirectness of evidence, Imprecision and Publication bias.**

We thank the reviewer for suggesting this important improvement to our manuscript.

**Minor comments:**

6. In general, the manuscript could benefit from professional English editing, as writing problems and incoherencies, especially regarding verb tense, currently exist.

**Response:**

We sincerely appreciate your valuable feedback regarding the language quality of our manuscript. We fully acknowledge that the writing required improvement in verb tense consistency and overall coherence. We have meticulously revised the manuscript. Should additional language editing be necessary, we will employ the journal's recommended editing service.

7. In the methods section, authors have mentioned both Medline and PubMed as databases. Please either remove one of these databases or amend the sentence to something like: “Medline via PubMed”.

**Response:**

We sincerely appreciate this precise technical advice. W**e have Changed the original wording to specify "Medline via PubMed".**

This modification has improved the technical accuracy of our methodology description. We thank the reviewer for bringing this important detail to our attention.

8. The authors could cite the two articles that could not be retrieved.

**Response:**

We thank the reviewer for this helpful suggestion. We have now properly cited the two articles that could not be retrieved in full text **[43,44].**

**[43]Vavilis D , Karavida A , Tzitzimikas S ,et al.Serum maternal carcinoembryonic antigen-125 (CA-125) levels in first trimester threatened abortion with demonstrable fetal heart activity[J].Middle East Fertility Society Journal, 2001, 6(2):159-162.**

**[44]Fiegler P ,Kazimierz Kamiński, Wegrzyn P .[Serum levels of CA-125 antigen during the first trimester of pregnancy complications and the risk of miscarriage].[J].Ginekologia Polska, 2003, 74(5):345.**

9. Table 1:
The utilized assay in each study for detecting CA125 could be added to Table 1.
Please use one form for synonymous words in Table 1 (venipuncture = venous blood sample).

**Response:**

Thank you for this suggestion. We have added a new column **(Utilized assay) in Table 1** specifying the CA125 detection method for each included study. We have standardized all terminology in Table 1, consistently **using "venipuncture" throughout**. 

10. Figure captions could use improvements; in their current form, they aren’t clear as standalone sentences.
e.g: Fig3 could be updated to: “Summary receiver operating curve and empirical bayes estimates for the value of CA-125 in pregnancy outcome prediction in women with threatened abortion”
Please update figure captions to be clear as a standalone sentence.

**Response:**

Thank you for your valuable comments and suggestions. We have carefully revised all figure captions to ensure they are clear and can stand alone as complete sentences, as recommended. Below is a summary of the changes made:

**Fig 1. Flowchart of study selection process for the systematic review and meta-analysis of CA125 in predicting miscarriage risk in patients with threatened abortion.**

**Fig 2. Risk of bias assessment of included studies evaluating CA125 for miscarriage risk prediction in threatened abortion, analyzed using the QUAPAS tool.**

**Fig 3. Summary receiver operating characteristic (SROC) curve and empirical Bayes estimation of CA125 as a prognostic biomarker for miscarriage risk prediction in threatened abortion.**

**Fig 4. Forest plot of meta-analysis evaluating the sensitivity and specificity of CA125 in predicting miscarriage risk in patients with threatened abortion.**

**Fig 5. Likelihood ratio quadrant distribution plot assessing the predictive performance of CA125 for miscarriage risk in patients with threatened abortion.**

**Fig 6. Meta-regression analysis identifying potential sources of heterogeneity in studies assessing CA125 for miscarriage risk prediction.**

**Fig 7. Deek's funnel plot assessing publication bias in the meta-analysis of CA125 in predicting miscarriage risk in patients with threatened abortion.**

Thank you again for your insightful feedback, which has improved the clarity of our manuscript.

12. Currently, the raw data necessary for duplicating the analysis are not presented in the manuscript, in contrast to the data availability statement. Please either provide raw data checklist or change the data availability statement.

**Response:**

Thank you for highlighting this important issue. **We have now included the raw data (including TP, FP, TN, and FN values for each study) as a new column in Table 1** to ensure full transparency and reproducibility.

This revision aligns with best practices for open data sharing and allows readers to verify our meta-analysis results.

13. Line 171. A verb seems to be missing after “remaining 22 were.”

**Response:**

Thank you for catching this oversight. We have revised the sentence to:"remaining 22 were **retained**"

Reviewer #2: Dear Authors thank you, please check the attached file
Dear Editor,
Thank you for the opportunity to review the manuscript. This is an updated systematic from “Role of serum biomarkers in the prediction of outcome in women with threatened miscarriage: a systematic review and diagnostic accuracy meta-analysis”. I'm not really sure what the clinical value of this is, some of the articles don't have precise inclusion and exclusion criteria. This factor is influenced by many items, such as VF, which is not mentioned in any of the articles. I have some comments:

Title
• The term “pregnancy outcomes” is broad and could mean different things, such as miscarriage, preterm labor, or live birth rates. A more specific definition of the outcomes being assessed would make the title clearer.

**Response:：**

We sincerely appreciate your valuable suggestion. As recommended, we have revised the title of the manuscript to:

**“Assessment of the prognostic value of CA125 for miscarriage risk in patients with threatened abortion—a systematic review and Meta-analysis”**

**We really appreciate your professional advice.**

Abstract
• The phrase "clinical value of serum CA125 levels" is not very precise. It would be assisted by specifying whether the focus is on the prediction of miscarriage, pregnancy outcomes in general, or a generalized diagnostic strategy.
• There is no discussion of why CA125 is being evaluated or its significance as a biomarker in threatened abortion. Briefly describing CA125's role in this context would improve accessibility for readers unfamiliar with the topic.
• The conclusion claims “high diagnostic value” for CA125 but does not link this to clinical decision-making or how it could change current practice. Adding a practical implication would strengthen the conclusion.

**Response:：**

1.We agree with you and have revised the abstract to explicitly state that the study focuses on predicting **miscarriage risk.**

2.We thank you for this suggestion. We have added a concise explanation of CA125’s biological relevance in the **BACKGROUND** **(Page 2, Lines 21–25).**

3.We fully agree and have revised the **CONCLUSION** to highlight clinical implications **(Page 3, Lines 48-54).**

We sincerely appreciate the reviewers' insightful suggestions.

Introduction
• While CA125 is introduced as a tumor marker, little is said about its physiological role in pregnancy and how it might be associated with risk of miscarriage. The reader is left to wonder why it is being measured.
• Please explain the physiologic and pathologic items that may rise ca125 in pregnancy.

**Response:：**

We thank you for this important observation.  In the revised manuscript **(Page 4 Lines 71-76),** we have supplemented the relevant introduction.

Method
• While the inclusion criteria for threatened abortion are well-described, they could benefit from referencing standardized definitions or guidelines, as variability in diagnostic criteria could introduce inconsistency.
• Whilst heterogeneity due to threshold and non-threshold effects is accounted for, there is no mention of how other sources of heterogeneity, such as study design and population differences, are dealt with.

**Response:：**

We thank you for this valuable suggestion. In the revised **Methods** section **(Page 5, Lines 87-88),** we have referenced the following standardized criteria:

**“ACOG Practice Bulletin No. 200: Early Pregnancy Loss.[J] .Obstet Gynecol, 2018, 132: 0.”**

We have also added the following clarify in **Statistical analysis (Page 5, Lines 144-145)**:

**“To investigate the potential sources of high heterogeneity unrelated to threshold effects, we conducted a meta-regression analysis.”**

Result
• he sensitivity and specificity results show significant heterogeneity (I²=56.43% for sensitivity, I²=81.82% for specificity). Although heterogeneity sources are explored via meta-regression, the discussion of how this impacts the reliability of pooled estimates is lacking.

**Response:：**

Thank you for your valuable suggestions. In the revised manuscript, also following Reviewer 1's suggestion, we have conducted **GRADE evidence assessments** for prognostic accuracy measures including **specificity and sensitivity.** As detailed in **Table 2,** the evidence certainty for both sensitivity and specificity were affected by heterogeneity.

• Although patients are from three continents, the specific proportions from each region are not detailed. Given that the meta-regression shows population differences significantly affect heterogeneity, this could undermine the generalizability of results. 8/13 of the articles are from Egypt. The results may not be very generalizable.

**Response:：**

We agree with your concern regarding population distribution. In the revised manuscript, we supplemented the specific proportions for each region in **Study characteristics (Page 9, Lines167-168)** and **Discussion (Page17, Lines286-287).** In addition, we added in the Meta-regression and Discussion that the results may not be generalizable because of population differences (**Page 12, Lines214-216; Page 16-17, Lines282-296)**.

Discussion
• Whereas the discussion indicates that most of the study population was African, it does not provide adequate context for how this unbalance might affect the results' generalizability. An elaboration on demographic differences would be enriching.

**Response:：**

We sincerely appreciate your insightful feedback. In the revised manuscript, we enriched the content by adding a discussion of population differences **(Page 16-17, Lines282-296).**

We appreciate your suggestion to strengthen this important component of our paper.

• While the manuscript notes variability in CA125 threshold values, it fails to address how this impacts clinical decision-making or propose solutions for standardization.

**Response:：**

We sincerely appreciate you for your valuable feedback regarding the discussion section of our manuscript. In the revised manuscript, we added relevant content (**Page 17, Lines300-304; Page 18, Lines308-315)**.

We believe these additions have significantly improved the manuscript by providing deeper insights into our results and their broader significance.

**.**
